# Sugar and Teeth

**Published:** 1868-12

**Authors:** 


### SUGAR AND TEETH.

In a previous number it was stated that pure sugar and candies, having no residue, could not, by lodgement about the teeth, injure them; and that if used in moderation, neither sugar nor candies were prejudicial to the teeth or health of young children or grown persons; that there was more or less sugar in all vegetable food; but as concentrations were liable to abuse, we advised that they should be taken at regular meals.

The *Medical Journal* of Charleston, S. C., states the conclusions of M. Larez:

1st. Refined Sugar injures teeth, either by immediate contact, or by gas developed in the stomach.

2d. That a tooth soaked in sugar water, becomes jelly like, from the sugar combining with the lime of the tooth.

To which the *Scientific American*, good authority in cogs and pulleys and piston-rods and all that, dogmatises thus: "The foregoing conclusions are correct, and candies and condiments should be avoided, especially by children. Maple Sugar renders the teeth sensitive."

The whole statement is based on the assertion, that a tooth put in a saturated solution of sugar becomes gelatinous. This is not denied. But it is no argument. The gastric juice begins to eat up the stomach, as soon as a man dies. But we know that the gastric juice has no injurious effect on a healthy living stomach. What injures a dead tooth, may have no effect on a living one. The argument from the living to the dead; from the hospital to the private-house; from the rich to the poor; from the tropics to the poles; from the healthy to the diseased; from animal phenomena in the natural state, to those presented when agonizing under the knife or virulent poisons, has strewn multitudes of delusions throughout the whole of medical literature. If an isolated case were worth anything, we can state for ourselves, that we ate all the sugar we could get while a child; and now, use "lasses" three times a day, and we think our teeth will compare favorably with those of any other person, on our side of forty-five. It is general ill-health which makes us toothless before our time, induced by over eating and under exercise, by hot bread, and late and large suppers. Away with your single hobbies, gentlemen. Widen your views.

